# Predicting microbiome compositions from species assemblages through deep learning

**DOI:** 10.1002/imt2.3

**Published:** 2022-03-01

**Authors:** Sebastian Michel‐Mata, Xu‐Wen Wang, Yang‐Yu Liu, Marco Tulio Angulo

**Affiliations:** ^1^ Center for Applied Physics and Advanced Technology Universidad Nacional Autónoma de México Juriquilla Mexico; ^2^ Department of Ecology and Evolutionary Biology Princeton University Princeton New Jersey USA; ^3^ Channing Division of Network Medicine, Department of Medicine Brigham and Women's Hospital and Harvard Medical School Boston Massachusetts USA; ^4^ CONACyT—Institute of Mathematics Universidad Nacional Autónoma de México Juriquilla Mexico

**Keywords:** deep learning, microbiome composition, species assemblage

## Abstract

Microbes can form complex communities that perform critical functions in maintaining the integrity of their environment or their hosts' wellbeing. Rationally managing these microbial communities requires improving our ability to predict how different species assemblages affect the final species composition of the community. However, making such a prediction remains challenging because of our limited knowledge of the diverse physical, biochemical, and ecological processes governing microbial dynamics. To overcome this challenge, we present a deep learning framework that automatically learns the map between species assemblages and community compositions from training data only, without knowing any of the above processes. First, we systematically validate our framework using synthetic data generated by classical population dynamics models. Then, we apply our framework to data from in vitro and in vivo microbial communities, including ocean and soil microbiota, *Drosophila melanogaster* gut microbiota, and human gut and oral microbiota. We find that our framework learns to perform accurate out‐of‐sample predictions of complex community compositions from a small number of training samples. Our results demonstrate how deep learning can enable us to understand better and potentially manage complex microbial communities.

## Highlights

- A deep learning framework was developed to predict community compositions from species assemblages.
- The framework does not require knowing any microbial dynamics.
- Validation in various data shows accurate predictions.

## Graphical abstract

A deep learning method to predict microbial compositions from species assembalges.

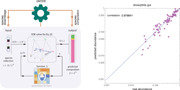

## INTRODUCTION

Microbes can form complex multispecies communities that perform critical functions in maintaining the integrity of their environment [1,2] or the well‐being of their hosts [3, 4, 5, 6]. For example, microbial communities play key roles in nutrient cycling in soils [7] and crop growth [8]. In humans, the gut microbiota plays important roles in our nutrition [9], immune system response [10], pathogen resistance [11], and even our central nervous system response [5]. Still, species invasions (e.g., pathogens) and extinctions (e.g., due to antibiotic administration) produce changes in the species assemblages that may shift these communities to undesired compositions [12]. For instance, antibiotic administrations can shift the human gut microbiota to compositions making the host more susceptible to recurrent infections by pathogens [13]. Similarly, intentional changes in the species assemblages, such as by using fecal microbiota transplantations, can shift back these communities to desired “healthier” compositions [14,15]. Therefore, improving our ability to rationally manage these microbial communities requires that we can predict changes in the community composition based on changes in species assemblages [16]. Building these predictions would also reduce managing costs, helping us to predict which changes in the species' assemblages are more likely to yield a desired community composition. Unfortunately, making such a prediction remains challenging because of our limited knowledge of the diverse physical [17], biochemical [18], and ecological [19,20] processes governing the microbial dynamics.

To overcome the above challenge, we present a deep learning framework that automatically learns the map between species assemblages and community compositions from training data only, without knowing the underlying microbial dynamics. We systematically validated our framework using synthetic data generated by classical ecological dynamics models, demonstrating its robustness to changes in the system dynamics and measurement errors. Then, we applied our framework to real data of both in vitro and in vivo communities, including ocean and soil microbial communities [21,22], *Drosophila melanogaster* gut microbiota [23], and human gut [24] and oral microbiota [25]. Across these diverse microbial communities, we find that our framework learns to predict accurate out‐of‐sample compositions given a few training samples. Our results show how deep learning can be an enabling ingredient for understanding and managing complex microbial communities.

## PREDICTING MICROBIOME COMPOSITIONS FROM SPECIES ASSEMBLAGES

Consider the pool Ω={1,…,N} of all microbial species (or taxa) that can inhabit an ecological habitat of interest, such as the human gut. A microbiome sample obtained from this habitat can be considered as a local community assembled from Ω with a particular *species assemblage*. The species assemblage of a sample is characterized by a binary vector $z\in {\left\{0,1\right\}}^{N}$, where its ith entry $z_{i}$ satisfies $z_{i}=1$ (or $z_{i}=0$) if the ith species is present (or absent) in this sample. Each sample is also associated with a *composition* vector $p\in \Delta^{N}$, where its ith entry $p_{i}$ is the relative abundance of the ith species, and $\Delta^{N}=\left\{p\in R_{\geq 0}^{N}|\sum_{i}p_{i}=1\right\}$ is the probability simplex. Therefore, our problem can be formalized as learning the map

(1) $$\varphi :z\in {\left\{0,1\right\}}^{N}⟼p\in \Delta^{N},$$

which assigns the composition vector p=φ(z) based on the species assemblage z. Note that the above map depends on many physical, biochemical, and ecological processes influencing the dynamics of microbial communities. These processes include the spatial structure of the ecological habitat [17], the chemical gradients of available resources [18], and inter/intraspecies interactions [20], among many others. Therefore, our limited knowledge of all these processes for most microbial communities renders the map of Equation (1) highly uncertain.

**Figure 1:**
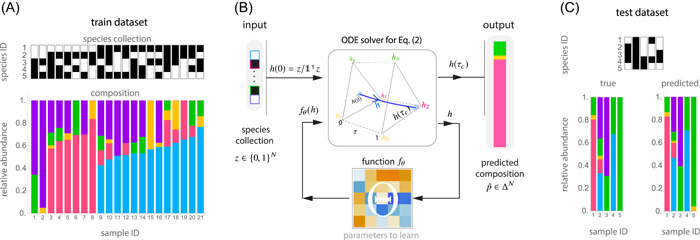
A deep learning framework to predict microbiome compositions from species assemblages. We illustrate this framework using experimental data from a pool of N=5 bacterial species in *Drosophila melangaster* gut microbiota [23]: *Lactobacillus plantarum* (blue), *Lactobacillus brevis* (pink), *Acetobacter pasteurianus* (yellow), *Acetobacter tropicalis* (green), and *Acetobacter orientalis* (purple). (A) We randomly split this data set into training ($D_{1}$) and test ($D_{2}$) data sets, which contain 80% and 20% of the samples, respectively. Each data set contains pairs (z,p) with the species assemblage $z\in {\left\{0,1\right\}}^{N}$ (top) and its corresponding composition $p\in \Delta^{N}$ (bottom) from each sample. (B) To predict compositions from species assemblages, our compositional neural ordinary differential equation (cNODE) framework consists of a solver for the ordinary differential equation shown in Equation (2), together with a chosen parametrized function $f_{\theta }$. During training, the parameters θ are adjusted to learn to predict the composition $\hat{p}\in \Delta^{N}$ of the species assemblage $z\in {\left\{0,1\right\}}^{N}$ in $D_{1}$. (C) After training, the performance is evaluated by predicting the composition of never‐seen‐before species assemblages in the test data set $D_{2}$. In this experimental microbiota, cNODE learned to perform accurate predictions of the composition in the test data set. For example, in the assemblage of species 3 and 4 (sample 26), cNODE correctly predicts that the composition is strongly dominated by a single species

Next, we show it is possible to predict the microbial composition from species assemblage without knowing the mechanistic details of all the above processes. Our approach consists in learning the map φ directly from a data set D with S microbiome samples. We arrange each of those samples as a pair (z,p) satisfying the map of Equation (1), see Figure 1A. Note that microbiome samples are readily available using standard metagenomic sequencing techniques.

### Conditions for predicting compositions from species assemblages

To ensure that the problem of learning φ from D is mathematically well‐posed, we make the following assumptions. First, we assume that the species pool in the habitat has universal dynamics [26] (i.e., different local communities of this habitat can be described by the same population dynamics model with the same parameters). This assumption is necessary because, otherwise, the map φ does not exist, implying that predicting community compositions from species assemblages has to be done in a sample‐specific manner, which is a daunting task. The universal dynamics assumption will be satisfied when samples in the data set were collected from similar environments. Indeed, in this case, the environmental factors can be treated as roughly fixed and hence need not be used for composition prediction. For in vitro communities, the universal dynamics assumption is satisfied if samples were collected from the same experiment or multiple experiments but with very similar environmental conditions. For in vivo communities, empirical evidence indicates that the human gut and oral microbiota of healthy adults, as well as certain environment microbiota, display strong universal dynamics [26].

Second, we assume that the compositions of the collected samples represent steady states of the microbial communities. This assumption is natural because the map φ is not well defined for highly fluctuating microbial compositions. We note that observational studies of host‐associated microbial communities such as the human gut microbiota indicate that they remain close to stable steady states in the absence of drastic dietary change or antibiotic administrations [24,27,28].

Finally, we assume that for each species assemblage $z\in {\left\{0,1\right\}}^{N}$ there is a unique steady‐state composition $p\in \Delta^{N}$. In particular, this assumption requires that true multistability does not exist for the species pool (or any subset of it) in this habitat. This assumption is required because, otherwise, the map φ is not injective, and the prediction of community compositions becomes mathematically ill‐defined.

In practice, we expect that the above three assumptions cannot be strictly satisfied. Therefore, any algorithm that predicts microbial compositions from species assemblages needs to be systematically tested to ensure its robustness against errors due to the violation of such approximations. Note that we can a priori check if a microbiome data set satisfies the universal dynamics assumption using the Dissimilarity‐Overlap analysis [26]. Yet, it is mathematically challenging to a priori check if the other two assumptions are satisfied for real data. Nevertheless, the ability to accurately predict microbiome compositions from species assemblage is a posteriori evidence of the validity of the above three assumptions.

### Learning to predict species compositions

Consider building a map ${\hat{\varphi }}_{\theta }:{\left\{0,1\right\}}^{N}\to \Delta^{N}$, parametrized by $\theta \in R^{p}$, giving the predicted composition $\hat{p}={\hat{\varphi }}_{\theta }\left(z\right)$ associated with the species assemblage z. Under the above assumptions, we can in principle learn the map of Equation (1) from the data set D by training ${\hat{\varphi }}_{\theta }$ (i.e., adjusting its parameters θ to ensure that ${\hat{\varphi }}_{\theta }$ approximates φ). Existing deep learning network architectures and training methods [29,30], such as ResNet [31] trained with a gradient descent algorithm, are natural candidates to solve this problem (Methods Section). We found that it is possible to train a ResNet architecture for predicting microbiome compositions in simple cases like small in vitro communities (Supporting Information Note S.2.1). But for large in vivo communities like the human gut microbiota, ResNet does not perform very well (Figure S1). The poor performance of ResNet is likely due to a vanishing gradient problem during training [32]. Namely, the ResNet architecture must satisfy two restrictions that are very particular to the map of Equation (1). First, the predicted compositions $\hat{p}$ must be compositional (i.e., $\hat{p}\in \Delta^{N}$). Second, the predicted relative abundance of any absent species in the assemblage must be identically zero (i.e., $z_{i}=0$ should imply that ${\hat{p}}_{i}=0$).

To overcome the limitations of traditional deep learning frameworks based on neural networks (such as ResNet) in predicting microbial compositions from species assemblages, we developed cNODE (compositional Neural Ordinary Differential Equation, see Methods Section and Figure 1B). We design the cNODE framework using the notion of Neural Ordinary Differential Equations, which can be interpreted as a continuous limit of ResNet architecture [33]. Crucially, the architecture and initialization of cNODE ensure that the above two restrictions are satisfied by construction. Furthermore, cNODE's architecture naturally circumvents the typical difficulties of handling zero values associated with compositional data analysis. Zero abundance values often occur in human microbiome datasets because of their highly personalized compositions across hosts (i.e., different individuals tend to have different species assemblages). To evaluate the prediction error of cNODE, one can choose any dissimilarity measure between the predicted and actual compositions related to a given species assemblage. Once this dissimilarity measure is selected, we train cNODE using a meta‐learning algorithm for a given number of epochs to minimize the average prediction error in a *training* data set $D_{1}$ (Methods Section). Using this meta‐learning algorithm improves the ability of cNODE for predicting the composition of never‐seen‐before species assemblages. Once trained, we evaluate the performance of cNODE by calculating its average prediction error in a *test* data set $D_{2}$ containing samples not used during the training.

Figure 1 illustrates the application of cNODE in a small experimental community of N=5 bacterial species of *Drosophila melanogaster* microbiota studied by Gould et al. [23]. The data set D obtained from this study has S=26 samples (Methods Section). To illustrate the potential of cNODE, we consider a training data set of 21 randomly chosen samples (Figure 1A). As explained before, we arrange each training sample as a pair of “species assemblage” z (top) and “composition” p (bottom). Once trained, the main use of cNODE is to predict the composition of “never‐seen‐before” species assemblages —namely, “test assemblages” that are not in the training data set. To evaluate the performance of cNODE for predicting such test assemblages, we use as test data set the remaining five experimental samples not included during training. Figure 1C shows that the trained cNODE predicts accurate compositions for the test species assemblages. For example, cNODE predicts that in the assemblage of species 3 with species 4 (which was not used for training), species 3 will become nearly extinct. This prediction agrees well with the actual experimental result (sample 26 in Figure 1C).

## RESULTS

### In silico validation of cNODE with large species pools

We first evaluated cNODE's performance using in silico microbiome samples generated as steady‐state compositions of pools with N=100 species and Generalized Lotka‐Volterra (GLV) population dynamics (Methods Section). We characterize the population dynamics of a species pool using two parameters. First, the *connectivity* C>0, characterizing how likely is that two species in the pool interact directly. Second, the *typical interaction strength* σ≥0, characterizing the typical effect of one species over the per‐capita growth rate of another species if they interact. Different habitats where the species pool is assembled are thus represented by different parameters (C,σ). Note that, despite its simplicity, the GLV model successfully describes the population dynamics of microbial communities in diverse environments, from the soil [39] and lakes [40] to the human gut [11,41,42].

**Figure 2:**
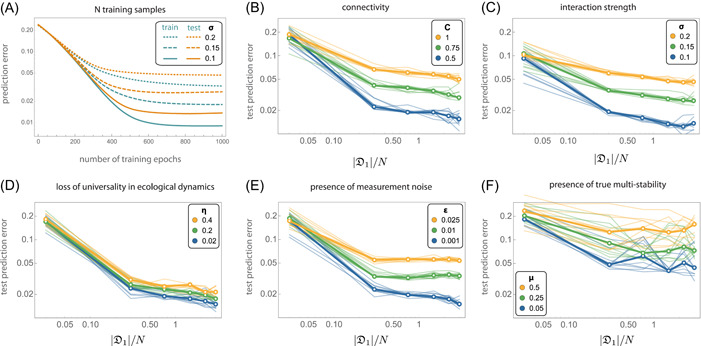
In silico validation of compositional neural ordinary differential equation (cNODE). Results are for pools of N=100 species with Generalized Lotka‐Volterra population dynamics (A–E) or population dynamics model with nonlinear functional responses that admits true multistability (F). The population dynamics is characterized by two parameters: the connectivity C>0 and the typical interaction strength σ≥0. In panels B–F, thin lines represent the prediction errors for 10 validations of training cNODE with a different data set. Mean errors are shown with thick lines. (A) Training cNODE using S=N samples with connectivity C=0.5 and different typical interaction strengths σ. (B) Performance of cNODE for in‐silico data sets with σ=0.1 and different connectivity C. (C) Performance of cNODE for in‐silico datasets with C=0.5 and different interaction strengths σ. (D) Performance of cNODE for in silico data sets with nonuniversal dynamics. The lack of universal dynamics is quantified by the value of η. For all datasets, σ=0.1 and C=0.5. (E) Performance of cNODE for in‐silico data sets with measurement errors quantified by ε. For all data sets, σ=0.1 and C=0.5. (F) Performance of cNODE for in‐silico data sets with multiple interior equilibria, quantified by the probability μ∈[0,1] of finding multiple equilibria. For all data sets, C=0.5,σ=0.1

Figure 2A shows the performance of cNODE during training. The training and test datasets have S=N samples for this panel. Note that the training prediction error decreases with the number of training epochs, especially for low values of σ. Interestingly, the test prediction error reaches a plateau after sufficient training epochs, regardless of the value of σ. This plateau implies that cNODE was adequately trained with low overfitting. Note that the plateau's value increases with σ (i.e., the test prediction error increases). This result remains valid for different training data set sizes and different values for the parameters (C,σ). In all these cases, the test prediction error reaches a plateau whose value increases both by increasing C (Figure 2B) or σ (Figure 2C). But, crucially, such an increase can be compensated by increasing the number of samples in the training data set. This result implies that, in general, cNODE requires a larger number of training samples in species pools with higher connectivity or higher typical interaction strength between species. Overall, these results suggest that using S=2N or more training samples is enough to adequately train cNODE, regardless of the habitat type. In this case, we also observe a high correlation between the true and predicted compositions in the test data set, as expected from a low test prediction error (Figure S2).

To systematically evaluate the robustness of cNODE against violation of its three key assumptions, we performed three types of validations. In the first validation, we generated datasets that violate the assumption of universal dynamics (Methods Section). In this case, if two species interact, the effect of one species over the per‐capita growth rate of the other species changes on average by η≥0 among samples in the data set. Therefore, the value η=0 corresponds to universal dynamics, and larger values of η correspond to more significant losses of universal dynamics. We find that cNODE is robust against universality loss as its asymptotic prediction error changes continuously and maintains a reasonably low test prediction error up to η=0.4 (Figure 2D). cNODE is also robust to losses of universal dynamics that occur when species interact with different species in a sample‐specific manner (Figure S3).

In the second validation, we evaluated the robustness of cNODE against measurement noises in the relative abundance of species (Methods Section). We characterize the noise intensity by a constant ε≥0. The measurement noise may cause some absent species to be measured as present and vice‐versa. We find that cNODE performs adequately up to ε=0.025 (Figure 2E).

In the final validation, we generated datasets with true multistability by simulating a population dynamics model with nonlinear functional responses (Methods Section). For each species assemblage, these functional responses generate two interior equilibria in different “regimes”: one regime with low biomass and the other with high biomass. Therefore, each species assemblage can have two associated compositions. We built training datasets by choosing a fraction (1−μ) of samples from the first regime and the rest from the second regime. We find that cNODE is robust enough to provide reasonable predictions up to μ=0.2 (Figure 2F).

### cNODE predicts microbiome compositions in real microbial communities

We evaluated cNODE using six microbiome datasets of different habitats (Supporting Information Note S4). The first data set consists of S=275 samples [43] of the ocean microbiome at the phylum taxonomic level, resulting in N=73 different taxa. The second data set consists of S=26 in vivo samples of *Drosophila melanogaster* gut microbiota of N=5 species [23], as described in Figure 1. The third data set has S=93 samples of in vitro communities of N=8 soil bacterial species [21]. The fourth data set contains S=113 samples of the Central Park soil microbiome [22] at the phylum level (N=36 phyla). The fifth data set contains S=150 samples of the human oral microbiome [25] at the genus level (N=73 genera). The final data set has S=106 samples of the human gut microbiome from the Human Microbiome Project [24] at the genus level (N=58 genera). Note that for each data set, to ensure cNODE has enough training samples, we chose to work at a specific taxonomic level so that the number of samples S≥2N, where N is the total number of taxa at the specific taxonomic level. Note that, based on the Dissimilarity‐Overlap analysis, all the six microbiome datasets display the signature of universal microbial dynamics to some extent (Supporting Information Note S4.5 and Figure S4).

**Figure 3:**
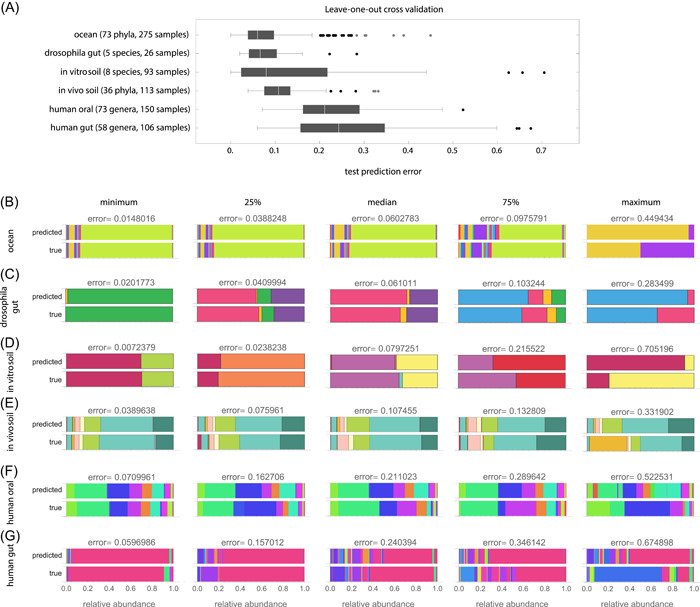
Predicting the composition of real microbiomes from species assemblages. Results of the compositional neural ordinary differential equation applied to six experimental microbial communities using a leave‐one‐out crossvalidation. (A) Prediction error obtained from a leave‐one‐out crossvalidation of each data set. (B–G) For each data set, we show the true and predicted compositions corresponding to the minimal prediction error, closer to the first quartile, median, closer to the third quartile, maximum prediction error (including outliers). All compositions shown in (B–G) are out‐of‐sample predictions

To evaluate cNODE, we performed the leave‐one‐out cross‐validation on each data set (Methods Section). The median test prediction errors were 0.06, 0.066, 0.079, 0.107, 0.211, and 0.242 for the six datasets, respectively (Figure 3A). These errors are consistent with the strength of universality observed in each data set. To understand the meaning of these errors, for each data set we inspected five pairs ($p,\hat{p}$) corresponding to the observed and out‐of‐sample predicted composition of five samples. We chose the five samples based on their test prediction error. Specifically, we selected those samples with the minimal error, close to the first quartile, closer to the median, closer to the third quartile, and with the maximal error (columns in Figure 3B–G, from left to right). We found that samples with errors below the third quartile provide acceptable predictions (left three columns in Figure 3B–G), while samples with errors close to the third quartile or with the maximal error do demonstrate salient differences between the observed and predicted compositions (right two columns in Figure 3B–G). Note that in the sample with largest error of the human gut data set (Figure 3G, rightmost column), the observed composition is dominated by *Prevotella* (pink) while the predicted sample is dominated by *Bacteroides* (blue). This drastic difference is likely due to different dietary patterns [44]. These results also confirm that 2N or more training samples are enough to adequately train cNODE, regardless of the habitat type. Note that using other taxonomic levels in these experimental datasets may change the performance of cNODE because it will effectively change the sample size.

## DISCUSSION

cNODE is a deep learning framework to predict microbial compositions from species assemblages only. We validated its performance using in silico, in vitro, and in vivo microbial communities, finding that cNODE learns to perform accurate out‐of‐sample predictions using a few training samples. Classic methods for predicting species abundances in microbial communities use inference based on population dynamics models [21,41,45,46]. However, these methods typically require high‐quality time‐series data of species absolute abundances, which can be difficult and expensive to obtain in vivo microbial communities. cNODE circumvents needing absolute abundances or time‐series data. However, compared to the classic methods, the cost to pay is that cNODE cannot be mechanistically interpreted because of the lack of identifiability inherent to compositional data [47,48]. We also found that cNODE can outperform existing deep‐learning architectures like ResNet, specially when predicting the composition of large in‐vivo microbiomes. Recently, Maynard et al. [49] proposed a statistical method to predict the steady‐state abundance in ecological communities [49]. This method requires absolute abundance data of species, which are not available in most microbiome datasets. cNODE can outperform this statistical method despite using only relative abundances (Supporting Information Note S6). See also Supporting Information Note S5 and Figure S5 for a discussion of how our framework compares to other related works.

Deep learning techniques are actively applied in microbiome research [50, 51, 52, 53, 54, 55, 56, 57, 58], such as for classifying samples that shifted to a diseased state [59], predicting infection complications in immunocompromised patients [60], or predicting the temporal or spatial evolution of certain species collection [61,62]. However, to the best of our knowledge, the potential of deep learning for predicting the effect of changing species assemblages was not explored nor validated before. Our framework, based on the notion of neural ODE [33], is a baseline that could be improved by incorporating additional information. For example, incorporating available environmental information such as pH, temperature, age, BMI, body‐site, and host's diet could enhance the prediction accuracy. This additional information would help us predict the species present in different environments. Adding “hidden variables” such as the unmeasured total biomass or unmeasured resources to our ODE will enhance the expressivity of the cNODE [63,64], but this may result in more challenging training. Finally, if available, knowledge of the genetic similarity between species can be leveraged into the loss function by using the phylogenetic Wasserstein distance [65] that provides a well‐defined gradient [66].

We anticipate that a useful application of our framework is to predict if by adding some species collection to a local community we can bring the abundance of target species below the practical extinction threshold. Thus, given a local community containing the target (and potentially pathogenic) species, we could use a greedy optimization algorithm to identify a minimal collection of species to add such that our architecture predicts that they will decolonize the target species.

Our framework has a few limitations. For example, cNODE cannot accurately predict the abundance of taxa that have never been observed in the training data set. An additional limitation of our current architecture is that it assumes that true multistability does not exist—namely, a community with a given species assemblage permits only one stable steady‐state, where each species in the collection has a positive abundance. For complex microbial communities such as the human gut microbiota, the highly personalized species collections make it very difficult to decide if true multistability exists or not. We could extend our framework to handle multistability by predicting a probability density function for the abundance of each species. True multistability would correspond to predicting a multimodal density function in such a case. Datasets with insufficient sequencing depth or coverage can produce samples with “fake” multistability, leading to prediction errors that our framework cannot resolve. Indeed, the in‐silico validation of cNODE in Figure 2 indicates that measurement errors can significantly degrade the performance of cNODE.

In conclusion, the many species and the complex, uncertain dynamics that microbial communities exhibit, have been fundamental obstacles in our ability to learn how they respond to alterations, such as removing or adding species. Moving this field forward may require losing some ability to interpret the mechanism behind their response. In this sense, deep learning methods could enable us to rationally manage and predict complex microbial communities' dynamics.

## METHODS

### A ResNet architecture for predicting microbiome compositions from species assemblages

As a top‐rated tool in image processing, ResNet is a cascade of L≥1 hidden layers where the state $h_{\ell }\in R^{N}$ of the ℓth hidden layer satisfies $h_{\ell }=h_{\ell -1}+f_{\theta }\left(h_{\ell -1}\right),\ell =1,\text{…},L$, for some parametrized function $f_{\theta }$ with parameters θ. These hidden layers are plugged to the input $h_{0}=g_{\;\text{in}\;}\left(z\right)$ and the output $\hat{p}=g_{\;\text{out}\;}\left(h_{L}\right)$ layers, where $g_{\;\text{in}\;}$ and $g_{\;\text{out}\;}$ are some functions. Crucially, for our problem, any architecture must satisfy two restrictions: (1) vector $\hat{p}$ must be compositional (i.e., $\hat{p}\in \Delta^{N}$); and (2) the predicted relative abundance of any absent species must be identically zero (i.e., $z_{i}=0$ should imply that ${\hat{p}}_{i}=0$). Simultaneously satisfying both restrictions requires that the output layer is a normalization of the form ${\hat{p}}_{i}=z_{i}h_{L,i}∕\sum_{j}z_{j}h_{L,j}$, and that $f_{\theta }$ is a non‐negative function (because $h_{L}\geq 0$ is required to ensure the normalization is correct). This result is likely due to the normalization of the output layer, which challenges the training of neural networks because of vanishing gradients [30]. The vanishing gradient problem is often solved by using other normalization layers such as the softmax or sparsemax layers [34]. However, we cannot use these layers because they do not satisfy the second restriction. We also note that ResNet becomes a universal approximation only in the limit L→∞, which again complicates the training [32].

### The cNODE architecture

In cNODE, an input species assemblage $z\in {\left\{0,1\right\}}^{N}$ is first transformed into the initial condition $h\left(0\right)=z∕1^{⊺}z\in \Delta^{N}$, where $1={\left(1,\text{…},1\right)}^{⊺}\in R^{N}$ (left in Figure 1B). This initial condition is used to solve the set of nonlinear ODEs

(2) $$\frac{\;dh\left(\tau \right)}{d\;\tau }=h\left(\tau \right)⊙\left[f_{\theta }\left(h\left(\tau \right)\right)-1\;h{\left(\tau \right)}^{⊺}\;f_{\theta }\left(h\left(\tau \right)\right)\right].$$

Here, the independent variable τ≥0 represents a virtual “time”. The expression h⊙v is the entry‐wise multiplication of the vectors $h,v\in R^{N}$. The function $f_{\theta }:\Delta^{N}\to R^{N}$ can be any continuous function parametrized by θ. For example, it can be the linear function $f_{\theta }\left(h\right)=\Theta h$ with parameter matrix $\Theta \in R^{N\times N}$ (bottom in Figure 1B), or a more complicated function represented by a feedforward deep neural network. Note that Equation (2) is a general form of the replicator equation—a canonical model in evolutionary game theory [35]—with $f_{\theta }$ representing the fitness function. By choosing a final integration “time” $\tau_{c}>0$, Equation (2) is numerically integrated to obtain the prediction $\hat{p}=h\left(\tau_{c}\right)$ that is the output of cNODE (right in Figure 1B). We choose $\tau_{c}=1$ without loss of generality, as τ in Equation (2) can be rescaled by multiplying $f_{\theta }$ by a constant. The cNODE thus implements the map

(3) $${\hat{\varphi }}_{\theta }:z\in {\left\{0,1\right\}}^{N}⟼\hat{p}\in \Delta^{N},$$

taking an input species assemblage z to the predicted composition $\hat{p}$ (see Supporting Information Note S.1 for implementation details). Note that Equation (2) is key to cNODE because its architecture guarantees that the two restrictions imposed before are naturally satisfied. Namely, $\hat{p}\in \Delta^{N}$ because the conditions $h\left(0\right)\in \Delta^{N}$ and $1^{⊺}\;dh∕d\;\tau =0$ imply that $h\left(\tau \right)\in \Delta^{N}$ for all τ≥0. Additionally, $z_{i}=0$ implies ${\hat{p}}_{i}=0$ because h(0) and z have the same zero pattern, and the right‐hand side of Equation (2) is entry‐wise multiplied by h.

### Training cNODE

We train cNODE by adjusting the parameters θ to approximate φ with ${\hat{\varphi }}_{\theta }$. To do this, we first choose a distance or dissimilarity measure d(p,q) to quantify how dissimilar are two compositions $p,q\in \Delta^{N}$. We choose the Bray‐Curtis [36] dissimilarity to present our results, however, the performance of cNODE is quite robust to the specific distance or dissimilarity measure used (Figure S6). Specifically, for a data set $D_{i}\subseteq D$, we use as loss function the *prediction error*

(4) $$E\left(D_{i}\right)=\frac{1}{|D_{i}|}\sum_{\left(z,p\right)\in D_{i}}d\left(p,{\hat{\varphi }}_{\theta }\left(z\right)\right).$$

Second, we randomly split the data set D into training $D_{1}$ and test $D_{2}$ datasets. Next, we choose an adequate functional form for $f_{\theta }$. In our experiments, we found that the linear function $f_{\theta }\left(h\right)=\Theta h,\Theta \in R^{N\times N}$, provides accurate predictions for the composition of in silico, in vitro, and in vivo communities. Importantly, despite $f_{\theta }$ is linear, the map ${\hat{\varphi }}_{\theta }$ is nonlinear because it is the solution of the nonlinear ODE of Equation (2). Finally, we adjust the parameters θ by minimizing Equation (4) on $D_{1}$ using a gradient‐based meta‐learning algorithm [37]. This learning algorithm enhances the generalizability of cNODE (Supporting Information Note S1.2 and Figure S1). Training cNODE with a data set of 100 species, 100 training samples, and 100 epochs takes about 30 min on a Linux machine with six Intel Xeon CPUs (E7‐4870 v2) @ 2.30 GHz.

Once trained, we calculate cNODE's test prediction error $E\left(D_{2}\right)$ that quantifies cNODE's performance in predicting the compositions of never‐seen‐before species assemblages. Test prediction errors could be due to a poor adjustment of the parameters (i.e., inaccurate prediction of the training set), low ability to generalize (i.e., inaccurate predictions of the test data set), or violations of our three assumptions (universal dynamics, steady‐state samples, no true multistability).

### Generating in‐silico data for validating cNODE

We generated in silico data for validating cNODE as steady‐state compositions of pools with N species and generalized Lotka‐Volterra (GLV) population dynamics. The GLV model reads [38]:

(5) $$\frac{\;dx_{i}\left(t\right)}{d\;t}=x_{i}\left(t\right)\left[r_{i}+\sum_{j=1}^{N}a_{ij}\;x_{j}\left(t\right)\right],i=1,\text{…},N.$$

Above, $x_{i}\left(t\right)$ denotes the abundance of the ith species at time t≥0. The GLV model has as parameters the interaction matrix $A=\left(a_{ij}\right)\in R^{N\times N}$, and the intrinsic growth‐rate vector $r=\left(r_{i}\right)\in R^{N}$. The parameter $a_{ij}$ denotes the inter‐ (if j≠i) or intra‐ (if j=i) species *interaction strength* of species j to the per‐capita growth rate of species i. The parameter $r_{i}$ is the intrinsic growth rate of species i. The interaction matrix A determines the ecological network G(A) underlying the species pool. Namely, this network has one node per species and edges (j→i)∈G(A) if $a_{ij}\neq 0$. The *connectivity* C∈[0,1] of this network is the proportion of edges it has compared to the $N^{2}$ edges in a complete network.

To validate cNODE, we generated synthetic microbiome samples as steady‐state compositions of GLV models with random parameters by choosing $a_{ij}\sim \;\text{Bernoulli}\left(C\right)\text{Normal}\;\left(0,\sigma \right)$ if $i\neq j,a_{ii}=-1$, and $r_{i}\sim \;\text{Uniform}\;\left[0,1\right]$, for different values of connectivity C and characteristic inter‐species interaction strength σ>0 (Supporting Information Note S3).

### Generating in silico data to test the robustness of cNODE

For this, given a “base” GLV model with parameters (A,r), we consider two forms of universality loss (Supporting Information Note S3). First, samples are generated using a GLV with the same ecological network but with those non‐zero interaction strengths $a_{ij}$ replaced by $a_{ij}+\;\text{Normal}\;\left(0,\eta \right)$, where η>0 characterizes the changes in the typical interaction strength. Second, samples are generated using a GLV with slightly different ecological networks obtained by randomly rewiring a proportion ρ∈[0,1] of their edges.

In the second validation, we evaluated the robustness of cNODE against measurement noises in the relative abundance of species. For this, for each sample p, we first change the relative abundance of the ith species from $p_{i}$ to $\max\left\{0,p_{i}+\;\text{Normal}\;\left(0,\varepsilon \right)\right\}$, where ε≥0 characterizes the measurement noise intensity. Then, we normalize the vector p to ensure it is still compositional, that is, $p\in \Delta^{N}$. Due to the measurement noise, some species that were absent may be measured as present and vice‐versa.

In the third validation, we generated datasets with true multistability by simulating a population dynamics model with nonlinear functional responses (Supporting Information Note S3). For each species collection, these functional responses generate two interior equilibria in different “regimes”: one regime with low biomass, and the other regime with high biomass. We then train cNODE with datasets obtained by choosing a fraction (1−μ) of samples from the first regime, and the rest from the second regime.

### Validating cNODE using real microbiome data sets

To validate cNODE, we performed a leave‐one‐out cross‐validation over real microbiome data sets (see descriptions on Supporting Information Note S4). For each data set, we measured the prediction error of cNODE using each sample as a test set and the rest of the samples as a training set. We repeated this procedure for different learning rates and mini‐batch sizes and selected the hyperparameters that minimized the average prediction error over the samples (see Table S1).

## CONFLICT OF INTERESTS

The authors declare that there are no conflict of interests.

## AUTHOR CONTRIBUTIONS

Marco Tulio Angulo and Yang‐Yu Liu conceived and designed the project. Sebastian Michel‐Mata did the numerical analysis. Sebastian Michel‐Mata and Xu‐Wen Wang performed the real data analysis. All authors analyzed the results. Marco T. Angulo and Yang‐Yu Liu wrote the manuscript. Sebastian Michel‐Mata and Xu‐Wen Wang edited the manuscript.

## Supporting information

Supplementary Information

## Data Availability

The data and code used in this study are available at https://github.com/michel-mata/cNODE.jl.
