# Supplementary material for: Predicting microbiome compositions from species assemblages through deep learning
Source: Imeta. 2022 Mar 1;1(1):e3. doi: 10.1002/imt2.3 (PMC9221840; doi:10.1002/imt2.3)
Supplement: Supplementary file 1 — Supplementary Information [file IMT2-1-e3-s001.pdf]

Predicting microbiome compositions from species assemblages through deep learning

Sebastian Michel-Mata[1,2], Xu-Wen Wang[3], Yang-Yu Liu[3*], and Marco Tulio Angulo[4,**]

[1]Center for Applied Physics and Advanced Technology, Universidad Nacional Autónoma de México, Juriquilla 76230, México.

[2]Department of Ecology and Evolutionary Biology, Princeton University, Princeton, NJ 08544, USA

[3]Channing Division of Network Medicine, Department of Medicine, Brigham and Women's Hospital and Harvard Medical School, Boston, Massachusetts 02115, USA.

[4]CONACyT - Institute of Mathematics, Universidad Nacional Autónoma de México, Juriquilla 76230, México.

To whom correspondence should be addressed: *yyl@channing.harvard.edu or **mangulo@im.unam.mx

Supplementary Notes

Contents

S1 Implementation of the compositional Neural Ordinary Differential Equation 1
S1.1 Flux implementation of cNODE. 1
S1.2 Training cNODE. 1

S2 Comparison of two deep learning frameworks in predicting microbiome compositions 2
S2.1 A ResNet architecture for predicting microbiome compositions. 2
S2.2 Performance comparison. 2

S3 Population dynamics for the *in silico* validations. 4
S3.1 Generating datasets with universal dynamics. 4
S3.2 Generating datasets with non-universal dynamics. 4
S3.3 Adding measurement noise to a dataset. 5
S3.4 Generating datasets with multi-stability. 5

S4 Description of the experimental datasets. 6
S4.1 In-vivo drosophila core gut microbiome. 6
S4.2 In-vitro soil community. 6
S4.3 In-vivo soil microbiome. 6
S4.4 Human gut microbiome. 6
S4.5 Universality of experimental datasets. 8

S5 Related work. 10

S6 Comparing cNODE with Maynard et al.'s method. 12

S1. Implementation of the compositional Neural Ordinary Differential Equation

S1.1 Flux implementation of cNODE.

We implemented cNODE using `Flux`[1], a library for machine learning in the Julia programming language with support for Neural Ordinary Differential Equations[2]. A complete implementation of cNODE is given in the file `cNODE.jl`.

Our implementation is based on a structure called `FitnessLayer` that contains cNODE's parameter $\Theta \in \mathbb{R}^{N \times N}$. This parameter is initialized using the Xavier's method[3]. When evaluated in a composition p, the `FitnessLayer` computes first $f_\theta(p) = \Theta p$. More complex functions f_θ can be easily incorporated in the code. Finally, the structure uses f_θ to calculate the right-hand side of the ODE in Eq. (**??**).

To predict the composition $\hat{p} \in \Delta^N$ associated with a species collection $z \in \{0, 1\}^N$, cNODE numerically solves such an the ODE in Eq.(2). In `Flux`, this is automated by the function `neural_ode`, which constructs the cNODE by building a NODE with the dynamics specified by the `FitnessLayer`.

The dynamics is numerically integrated over the interval $\tau \in [0, \tau_c]$ using the `Tsit5` method[4], which is the default integration method for nonstiff ODEs in Julia. We choose $\tau_c = 1$ without loss of generality, as τ in Eq. (**??**) can be rescaled by multiplying f_θ by a constant. After integration, the final value at time $\tau = 1.0$ is returned as the prediction of cNODE.

The loss function calculates the average Bray-Curtis dissimilarity between there true composition $p \in \Delta^N$ and the prediction $\hat{p} \in \Delta^N$ generated by cNODE.

S1.2 Training cNODE.

To train cNODE, we adjust the parameters θ to minimize the loss over the training set. We experimented with two training algorithms:

1. **The ADAM algorithm**[5]. ADAM is a widely used gradient-based stochastic optimization algorithm that often compares favorably to other gradient optimization algorithms[6]. We refer to [5, 6] for additional details.

2 **ADAM plus a first-order gradient-based meta-learning algorithm based on Reptile**[7]. In the meta-learning framework, lets consider a set $\mathcal{T}$ of different *tasks* that the network needs to perform, such as learning to predict in different datasets. Reptile works by sampling some task $\tau \in \mathcal{T}$, training on it to obtain the weights $\tilde{\theta}$, and then updating the initial weights θ towards $\tilde{\theta}$.

   To train cNODE, we used ADAM plus Reptile training[7]. More precisely, we defined each task as a random partition of the training set in mini-batches. In this way, training enhances cNODE's generalizing ability from the predictions regardless of any specific partition or any

sequence order of mini-batches. The algorithm we used is described below:

Algorithm 1: ADAM + Reptile for cNODE

Initialize θ, the vector of initial parameters
for *epoch = 1,2...* **do**
  Random partition of training set ρ
  Compute $\tilde{\theta} = \text{ADAM}_\rho^k(\theta)$, denoting k steps
  Update $\theta \leftarrow \theta + \text{ADAM}(\tilde{\theta} - \theta)$
end

Our implementation in the Julia language of the above algorithm is based on the model zoo of the library Flux[8].

S2. Comparison of two deep learning frameworks in predicting microbiome compositions

S2.1 A ResNet architecture for predicting microbiome compositions.

We tested the performance the classical ResNet architecture[2] for predicting microbiome compositions. More precisely, we used the input layer

$$h_0 = W_0 z + b_0,$$

where $W_0 \in \mathbb{R}^{N\times N}$ and $b_0 \in \mathbb{R}^N$ are parameters to adjust. We used $L = 3$ hidden layers of the form:

$$h_\ell = h_{\ell-1} + \text{ReLU}(W_{\ell-1}h_{\ell-1} + b_{\ell-1}), \quad \ell = 1, \cdots, L,$$

where $W_\ell \in \mathbb{R}^{N\times N}$ and $b_\ell \in \mathbb{R}^N$ are parameters to adjust. Finally, the output layer takes the form

$$\hat{p} = \left(\frac{h_{L,1}z_1}{\sum_j h_{L,j}z_j}, \cdots, \frac{h_{L,N}z_N}{\sum_j h_{L,N}z_N} \right).$$

A `Flux` implementation of this ResNet can be found in the file `ResNet.jl`.
We trained this ResNet architecture using the two methods described in S1.2.

S2.2 Performance comparison.

We compared the performance of cNODE and ResNet architecture for predicting the composition of four out of six real microbiome datasets described in Supplementary Note S4. These datasets contain a small set of species (between 5 and 58) and samples (between 26 and 113), allowing us to make computationally expensive leave-one-out cross validation analysis. Here, we also compared the effect of training both architectures with ADAM, and with ADAM plus the Reptile like metalearning algorithms (see Table.S1 for the training hyperparameters used for the cNODE and ResNet).

To perform the comparison we used a leave-one-out cross validation on each of the four datasets (Supplementary Fig. S1). From these results, some remarks are in order:

1. For both *in-vivo* datasets, cNODE trained with Reptile outperforms all other algorithms.

2. The ResNet architecture trained using only ADAM can provide reasonably accurate prediction for simple small species collections (i.e., for the $N = 5$ species in Drosophila gut and the $N = 8$ species in the soil *in-vitro* community).

3. For cNODE, training using the Reptile metalearning algorithm decreases the prediction error in the test dataset. Interestingly, using Reptile does not alway decreases the prediction error in the train dataset. Therefore, the Reptile metalearning algorithm is performing as desired to enhance the generalizing ability of cNODE.

4. For ResNet, training with the Reptile metalearning algorithm can increase the prediction errors in both the training and test datasets when compared to training only with ADAM.

5. The ResNet architecture exhibits a higher variability in the training set when compared to cNODE. This suggests that the performance of ResNet is significantly influenced by the initialization parameters. In particular, training a ResNet with Reptile can significantly increase the variability of prediction errors (see, e.g., the soil *in vitro* dataset).

Overall, the above remarks indicate that cNODE training with Reptile outperforms the other architectures when predicting complex microbial communities like human gut microbiota or *in vivo* soil communities.

Dataset	Inner Learning Rate	Outer Learning Rate	Minibatch size
Drosophila Gut	0.01	0.1	10
Soil in Vitro	0.01	0.1	5
Soil in Vivo	0.001	0.01	10
Human Gut	0.001	0.005	10
Human Oral	0.001	0.005	10
Ocean	0.01	0.025	10
GLV	0.001	0.0025	10

Supplementary Table S1: Training hyperparameters used for the cNODE and ResNet.

S3. Population dynamics for the *in silico* validations.

We generated *in-silico* species pools using the Generalized Lotka-Volterra (GLV) equations, a classical population dynamics model successfully applied to diverse microbial communities, from soils[9] and cheese[10], to the human body[11,12]. The GLV model takes the form

$$\frac{\mathrm{d}x(t)}{\mathrm{d}t} = x(t) \odot [Ax(t) + r], \quad x(0) = x_0, \tag{S1}$$

where $x(t) = (x_1(t), \cdots, x_N(t))^\top \in \mathbb{R}_{\geq 0}$ and $x_i(t)$ denotes the absolute abundance of species i at time t. Above, $x \odot v$ is the entry-wise multiplication of vectors $x, v \in \mathbb{R}^N$. The GLV model has two parameters: the interaction matrix of the species pool $A = (a_{ij}) \in \mathbb{R}^{N \times N}$, and the intrinsic growth-rate of the species $r = (r_i) \in \mathbb{R}^N$. In particular, the j-th species has a positive impact on the i-th species if $a_{ij} > 0$, a negative impact if $a_{ij} < 0$, and no impact if $a_{ij} = 0$. Recall also that the interaction matrix determines the underlying ecological network $\mathcal{G}(A)$ of the species pool. This network has one vertex associated to each species and an edge $(j \to i) \in \mathcal{G}(A)$ if $a_{ij} \neq 0$.

To generate the relative abundance vector $p \in \Delta^N$ corresponding to a local community with species collection $z \in \{0, 1\}^N$, we follow four steps:

1. Set the parameters (A, r).

2. Set the initial abundance of species $x_0 \in \mathbb{R}^N_{\geq 0}$ as

$$x_{0,\,i} = \begin{cases} 0 & \text{if } z_i = 0, \\ \text{Uniform}[0,1] & \text{otherwise,} \end{cases}$$

   for $i = 1, \cdots, N$.

3. Numerically integrate Eq. (S1) with initial condition x_0 until the system reaches a steady-state abundance $x^* = (x_1^*, \cdots, x_N^*) \in \mathbb{R}^N$. For the results presented in our paper, we choose a final integration time $t_f = 1000$.

4. Compute the relative abundance vector $p = (p_1, \cdots, p_N)^T \in \Delta^N$ as $p_i = x_i^* / \sum_j x_j^*$.

Using the above procedure, we generated a dataset $\mathfrak{D}$ by randomly sampling species collections $z \in \{0, 1\}^N$ and calculating the corresponding $\hat{p} \in \Delta^N$. Below we detail the construction of the three types of datasets used in the Main Text.

S3.1 Generating datasets with universal dynamics.

To generate a dataset with universal dynamics, we considered that all species collections have the same parameters (A, r). These parameters were generated as follows. The interaction matrix A of the community is obtained as the adjacency matrix of a directed weighted Erdös-Rényi random network with connectivity $C \in [0, 1]$. The edge-weights were chosen from a Normal distribution with zero mean and variance σ^2, where $\sigma > 0$ represents the "characteristic" inter-species interaction strength. The intrinsic growth r_i is chosen uniformly at random from the interval $[0, 1]$.

S3.2 Generating datasets with non-universal dynamics.

To generate a dataset with non-universal dynamics, we considered two possible sources for non-universality. First, the mechanisms of interaction between species may differ across local communities. In Eq. (S1), this translates as using different parameters a_{ij} for each non-zero interaction in

different local communities. Thus, in this case we replaced each $a_{ij} \neq 0$ by $a_{ij} + \eta\text{Normal}(0, 1)$, where $\eta > 0$ quantifies the *changes in the typical interaction strength*, and hence the "loss" of universality in this case.

Second, we considered that each local community may have a different ecological network. To model this case, we considered that the ecological network of each local community is obtained by *randomly rewiring* a proportion $\rho \in [0, 1]$ of the edges of a baseline ecological network $\mathcal{G}(A)$, thus shuffling a proportion of entries of the associated A matrix. Since $\rho = 0$ corresponds to universal dynamics, the magnitude of ρ quantifies the "loss" of universality in this case.

S3.3 Adding measurement noise to a dataset.

For a pair (z, p) in a dataset $\mathfrak{D}$, we added noise by replacing p_i by first adding a small noise $w_i = \max\{0, p_i + \varepsilon\text{Normal}(0, 1)\}$, and then normalizing to obtain the noisy measurement $p_i \leftarrow w_i / \sum_j w_j$. Here, the parameter $\varepsilon > 0$ controls the *measurement noise intensity*.

S3.4 Generating datasets with multi-stability.

To generate a dataset with true multi-stability, we calculated the steady-states from a population dynamics model with the following non-linear functional response:

$$\frac{\mathrm{d}x_i(t)}{\mathrm{d}t} = x_i(t)\left[r_i + \sum_{j=1}^{N} a_{ij}\,\frac{x_j(t)}{1 + h\,x_j(t)^2}\right], \quad i = 1, \cdots, N, \tag{S2}$$

where h denotes the handling time.

To generate steady-states with multi-stability, we first select a GLV model with a linear functional response (Eq. S1) and universal dynamics (Supplementary Notes S3), and compute the steady-state ξ^*. Note that the steady-state abundances satisfies the equation

$$r_i = -\sum_{j=1}^{N} a_{ij}\,\xi_j^*. \tag{S3}$$

The steady-states of Eq.(S2) satisfies the equation

$$\sum_{j=1}^{N} a_{ij}\,\frac{x_j}{1 + h\,x_j^2} + r_i = 0, \tag{S4}$$

so that we substitute Eq.S3 in Eq.S4 and solve for x_j the following quadratic equation:

$$h\xi_j^* x_j^2 - x_j + \xi_j^* = 0, \tag{S5}$$

for all j, and then we compute the relative abundance vector $p = (p_1, \cdots, p_N)^T \in \Delta^N$ as $p_i = x_i^* / \sum_j x_j^*$. To ensure that there are two real solutions for x_j, we chose $h = \frac{1}{4\xi_k^{*2}} > 0$ for some k.

The two steady-state abundances corresponds to a high and low total biomass regimes, respectively. To build the datasets, we chose a fraction (1-μ) from the first regime, and the rest from the second.

S4. Description of the experimental datasets.

S4.1 In-vivo drosophila core gut microbiome.

The drosophila dataset[13] contains the absolute abundance of the five species in each possible local community with different species collection. See Supplementary Table S2 for species IDs. There is five replicates for each of those species collections. We averaged those five replicates, discarded samples with a single species, and obtained the relative abundance of each of the remaining samples. This yielded 31 samples with different species collection.

ID	Genus	Species
1	Lactobacillus	plantarum
2	Lactobacillus	brevis
3	Acetobacter	pasteurianus
4	Acetobacter	tropicalis
5	Acetobacter	orientalis

Supplementary Table S2: Species IDs Drosophila gut microbiota.

S4.2 In-vitro soil community.

This laboratory community of $N = 8$ heterotrophic soil-dwelling bacterial species[14] described in Table S3. The available dataset contains 98 samples, including all solos, all duos, some trios, one septet and one octet.

ID	Genus	Species
1	Enterobacter	aerogenes
2	Pseudomonas	aurantiaca
3	Pseudomonas	chlororaphis
4	Pseudomonas	citronellolis
5	Pseudomonas	fluorescens
6	Pseudomonas	putida
7	Pseudomonas	veronii
8	Serratia	marcescens

Supplementary Table S3: Species IDs the in-vitro soil community.

S4.3 In-vivo soil microbiome.

The soil dataset consists of soil microbiome across Central Park in New York City consist of 1160 samples. This data set is 16S rRNA gene-based with variable region V4. The data is available at https://qiita.ucsd.edu/ under study ID 2140 and the detailed description of this data set can be found in Ref. [15]. We used the function `summarize_taxa.py` QIIME 1 to summarize taxa to different taxonomic levels with defaults options. Supplementary Table S4 provides the IDs associated to each phylumm.

S4.4 Human gut microbiome.

A 16S rRNA gene-based data set from variable regions V3 to V5. The data are available at http://www.hmpdacc.org/HMQCP/. We selected the samples from the stool body site. For multiple samples from the same subject, we only keep one single sample of that subject. To guarantee

ID	Kindgom	Phylum
1	Archaea	Crenarchaeota
2	Archaea	Euryarchaeota
3	Archaea	Parvarchaeota
4	Bacteria	others
5	Bacteria	Acidobacteria
6	Bacteria	Actinobacteria
7	Bacteria	Aquificae
8	Bacteria	Armatimonadetes
9	Bacteria	BHI80-139
10	Bacteria	Bacteroidetes
11	Bacteria	Chlorobi
12	Bacteria	Chloroflexi
13	Bacteria	Cyanobacteria
14	Bacteria	Elusimicrobia
15	Bacteria	FBP
16	Bacteria	Firmicutes
17	Bacteria	GN02
18	Bacteria	Gemmatimonadetes
19	Bacteria	Lentisphaerae
20	Bacteria	NC10
21	Bacteria	Nitrospirae
22	Bacteria	OD1
23	Bacteria	OP3
24	Bacteria	OP9
25	Bacteria	Planctomycetes
26	Bacteria	Proteobacteria
27	Bacteria	SBR1093
28	Bacteria	Spirochaetes
29	Bacteria	TM6
30	Bacteria	TM7
31	Bacteria	TPD-58
32	Bacteria	Tenericutes
33	Bacteria	Verrucomicrobia
34	Bacteria	WPS-2
35	Bacteria	WS1
36	Bacteria	WS6

Supplementary Table S4: Phylum IDs for soil dataset.

the model can be trained sufficiently, we summarized the taxa into the genus level and removed the genus with fewer than 50 reads. See also Supplementary Table S5 for genus ID.

S4.5 Universality of experimental datasets.

To assess the university of microbial dynamics, we performed the dissimilarity-overlap analysis[16]. For any pair of microbiome samples, the overlap is defined as half of the sum of relative abundances of the shared species of the two samples, while the dissimilarity is defined between the renormalized abundance profiles of the shared species of the two samples. For each of real dataset, we calculated the overlap and dissimilarity of all the sample pairs, represented by the dots in the dissimilarity-overlap plane. The negative correlation between dissimilarity and overlap in the high-overlap regime is a strong signal of universal microbial dynamics. We found that those real datasets all display signal of universal microbial dynamcis to some extent. The signal is strongest for the ocean microbiome, where a very strong negative relationship between dissimilarity and overlap can be seen in the high-overlap regime. This result is also consistent with the outstanding performance of cNODE in the ocean microbime dataset (as shown in Fig. **??**) of the main text.

ID	Phylum	Class	Order	Family	Genus
1	Firmicutes	Clostridia	Clostridiales	Veillonellaceae	*Veillonella*
2	Firmicutes	Clostridia	Clostridiales	Ruminococcaceae	*Clostridium*
3	Firmicutes	Clostridia	Clostridiales	Ruminococcaceae	*Bacteroides*
4	Tenericutes	Erysipelotrichi	Erysipelotrichales	Erysipelotrichaceae	*Coprobacillus*
5	Firmicutes	Clostridia	Clostridiales	ClostridialesFamilyXIII.IncertaeSedis	
6	Bacteroidetes	Bacteroidia	Bacteroidales	Porphyromonadaceae	*Odoribacter*
7	Firmicutes	Clostridia	Clostridiales	Lachnospiraceae	*Lachnobacterium*
8	Verrucomicrobia	Verrucomicrobiae	Verrucomicrobiales	Verrucomicrobiaceae	*Akkermansia*
9	Bacteroidetes				
10	Proteobacteria	Gammaproteobacteria	Pasteurellales	Pasteurellaceae	*Haemophilus*
11	Firmicutes	Clostridia	Clostridiales	Veillonellaceae	*Megasphaera*
12	Firmicutes	Bacilli	Lactobacillales	Streptococcaceae	*Streptococcus*
13	Firmicutes	Clostridia	Clostridiales	Ruminococcaceae	*Anaerotruncus*
14	Bacteroidetes	Bacteroidia	Bacteroidales	Porphyromonadaceae	*Parabacteroides*
15	Firmicutes	Clostridia	Clostridiales	Dehalobacteriaceae	*Dehalobacterium*
16	Tenericutes				
17	Firmicutes	Clostridia	Clostridiales	Lachnospiraceae	*Ruminococcus*
18	Firmicutes	Clostridia	Clostridiales	Lachnospiraceae	*Dorea*
19	Tenericutes	Erysipelotrichi	Erysipelotrichales	Erysipelotrichaceae	*Catenibacterium*
20	Proteobacteria	Deltaproteobacteria	Desulfovibrionales	Desulfovibrionaceae	*Desulfovibrio*
21	Firmicutes	Clostridia	Clostridiales	Ruminococcaceae	*Oscillospira*
22	Firmicutes	Clostridia	Clostridiales	Lachnospiraceae	*Clostridium*
23	Proteobacteria	Gammaproteobacteria	Enterobacteriales	Enterobacteriaceae	*Escherichia*
24	Firmicutes	Bacilli	Turicibacterales	Turicibacteraceae	
25	Firmicutes	Clostridia	Clostridiales	Ruminococcaceae	
26	Actinobacteria	Actinobacteria	Coriobacteriales	Coriobacteriaceae	*Collinsella*
27	Firmicutes	Clostridia	Clostridiales	Veillonellaceae	*Acidaminococcus*
28	Firmicutes	Clostridia	Clostridiales	ClostridialesFamilyXIII.IncertaeSedis	*Eubacterium*
29	Firmicutes	Bacilli	Lactobacillales	Lactobacillaceae	*Lactobacillus*
30	Firmicutes	Clostridia	Clostridiales	Lachnospiraceae	*Roseburia*
31	Firmicutes	Clostridia	Clostridiales	Ruminococcaceae	*Eubacterium*
32	Firmicutes	Clostridia	Clostridiales	Lachnospiraceae	*Lachnospira*
33	Bacteroidetes	Bacteroidia	Bacteroidales		
34	Proteobacteria	Deltaproteobacteria	Desulfovibrionales	Desulfovibrionaceae	*Bilophila*
35	Bacteroidetes	Bacteroidia	Bacteroidales	Prevotellaceae	*Prevotella*
36	Bacteroidetes	Bacteroidia	Bacteroidales	Rikenellaceae	*Alistipes*
37	Firmicutes	Clostridia	Clostridiales	Veillonellaceae	*Dialister*
38	Firmicutes	Clostridia	Clostridiales	Ruminococcaceae	*Ruminococcus*
39	Proteobacteria	Betaproteobacteria	Burkholderiales		
40	Tenericutes	Erysipelotrichi	Erysipelotrichales	Erysipelotrichaceae	*Holdemania*
41	Fusobacteria	Fusobacteria	Fusobacteriales	Fusobacteriaceae	*Fusobacterium*
42	Firmicutes	Clostridia	Clostridiales	Lachnospiraceae	*Eubacterium*
43	Firmicutes	Clostridia	Clostridiales	Ruminococcaceae	*Subdoligranulum*
44	Firmicutes	Clostridia	Clostridiales	Ruminococcaceae	*Faecalibacterium*
45	Tenericutes	Erysipelotrichi	Erysipelotrichales	Erysipelotrichaceae	*Clostridium*
46	Proteobacteria	Betaproteobacteria	Burkholderiales	Alcaligenaceae	*Sutterella*
47	Others				
48	Firmicutes	Clostridia	Clostridiales	Clostridiaceae	*Clostridium*
49	Firmicutes	Clostridia	Clostridiales	Lachnospiraceae	*Coprococcus*
50	Firmicutes	Clostridia	Clostridiales	Lachnospiraceae	*Blautia*
51	Bacteroidetes	Bacteroidia	Bacteroidales	Bacteroidaceae	*Bacteroides*
52	Firmicutes	Clostridia	Clostridiales	Clostridiaceae	
53	Firmicutes	Clostridia	Clostridiales	Lachnospiraceae	*Bacteroides*
54	Firmicutes	Clostridia	Clostridiales	Veillonellaceae	*Phascolarctobacterium*
55	Firmicutes	Clostridia	Clostridiales		
56	Actinobacteria	Actinobacteria	Bifidobacteriales	Bifidobacteriaceae	*Bifidobacterium*
57	Firmicutes	Clostridia	Clostridiales	Veillonellaceae	*Megamonas*
58	Firmicutes	Clostridia	Clostridiales	Lachnospiraceae	

Supplementary Table S5: Genus IDs for the human gut microbiota dataset.

S5. Related work.

Here we describe related methods, emphasizing some key differences with respect to our framework.

1. **Abundance prediction based on inferred population dynamics.** A classical method to predict species abundance in microbial ecosystems is modeling their population dynamics[10,11,12,14,17]. Typically, the model is a set of parametrized ODEs —such as the Generalized Lotka-Volterra equations— describing the changes over time in the *absolute* abundances of a set of species. The model is fitted to available temporal data of absolute abundance to infer parameters, such as intrinsic growth rates, inter-species interaction strengths, etc. Then, to predict the abundance of certain species collection, the fitted ODE model is solved starting from suitable initial condition. However, applying this method for large microbial communities like the human gut is challenging if not impossible because: 1) the absence of high-quality temporal data; and 2) typically only relative abundance of species are measured. Furthermore, because the very broad population dynamics that ecosystems display even at the scale of two species[18], it is always very challenging to choose an adequate parametrized ODE model for the population dynamics of the community.

2. **Predictions based on neural networks methods.** Larsen et al.[19] employed an artificial neural network to predict the *temporal* evolution of the composition of bacterial communities with a constant species collection. More precisely, they developed a bioclimatic model of relative microbial abundance that specifically incorporates interactions between biological units. They modeled the complex interactions between microbial taxa and their environment as an artificial neural network (ANN). This method is based on two key assumptions: (1) community patterns share mathematically describable relationships with environmental conditions; and (2) the ecosystem maintains a persistent microbial community. Note that the second assumption implies that this method can not be used to predict the impact of changing the species collections. Compared to method based on inferring population dynamics, this method has the advantage of not requiring to specify any model for the community dynamics. However, in contrast to our framework, this method cannot predict the effect of changing the species collection.

   Similarly, the recent work of Zhou et al.[20] uses a neural network to predict the temporal and *spatial* evolution of the composition of microbial communities with a constant species collection. More precisely, the authors formulated the prediction of microbial communities at unsampled locations as a multi-label classification task, where each location is considered as an instance and each label represents a microbe species. Based on a set of heterogeneous features extracted from the urban environment, they aimed to predict the presence or absence of a list of microbes species at a nearby location. Note, however, that this method cannot be immediately used to predict the effect of changing the species collection.

3. **Predictions based on statistical methods.** Recently, Maynard et al.[21] proposed a statistical method to predict species abundances from species collections. More precisely, based on measuring the absolute abundance of species at some steady-states of the ecosystem, this method assumes a linear model to predict all other steady-states. For the method to be applicable, it requires that the following assumptions are satisfied: (1) each species must be present in at least n distinct endpoints, not counting replicates; (2) each species must co-occur with each other species in at least one endpoint (that is, for every pair of species i and j, there must be some endpoint where i and j co-occur, possibly along with other

species); and (3) for each i there must exist a perfect matching between the n species and the endpoints in which they co-occur with i. As explained in the original manuscript[21], conditions (1) and (2) above requires that "coexistence among species must be reasonably widespread for [these] conditions to hold." This method may be challenging to apply for microbial communities because it requires measuring absolute abundances. Furthermore, because microbial communities tend to have nonlinear behaviors even at the scale of two species[18,22], the implicit assumption of linearity may fail to be satisfied. Finally, we note that cNODE does not require any of the above three assumptions to be applicable, although its prediction accuracy may be influenced by them.

Similarly, Tung et al.[23] use a linear regression method to predict species compositions from information of social networks of individuals. More precisely, the authors fit a classical linear mixed model to predict the relative abundance of species in a sample based on the following predictors: social group membership, age, sex, and read depth. We note here that the predictors in this approach are completely different than the predictors used in cNODE (i.e., it only uses species collections), and thus are not comparable.

S6. Comparing cNODE with Maynard et al.'s method.

Here we compare the performance of cNODE with the method of Maynard et al. for predicting endpoints[21]. This last method is described with details in item 3 of Supplementary Note S5.

To perform the comparison, we generated *in silico* datasets of $N = 5$ species with Generalized Lotka-Volterra dynamics. More precisely, we generated datasets with universal dynamics, maintaining the connectivity $C = 0.5$ constant and changing the typical interaction strength as $\sigma \in \{0.1, 0.2, 0.3, 0.4\}$. For each value of σ, we generated 10 datasets containing all $S = 2^5 - 1$ samples with different species collections following the simulation method described in Supplementary Note S3. We repeated this simulation method three times, obtaining three repetitions for the abundance of each species collections that can be used in Maynard's method. Additionally, because Maynard's method requires absolute abundances, we kept in the datasets both the absolute abundance and relative abundance of each steady-state that is reached. Using these datasets, we constructed training datasets by randomly choosing 70% of the samples, and the rest of the samples as test datasets.

To adjust Maynard's method we used the default parameters that were selected for $N = 4$ species. After this, we obtained the corresponding absolute abundance predictions of the test dataset. Finally, to allow a comparison with cNODE that predicts relative abundances, we transformed each predicted absolute abundance into a predicted relative abundance, and then calculated the prediction error using the Bray-Curtis dissimilarity.

For cNODE, we used the exact same training dataset as for Maynard's method, the only difference being that we trained cNODE with relative abundances. We choose a inner learning rate of 0.001, an outer learning rate of 0.005, and trained cNODE for 500 epochs using mini batches of 5 samples. We then calculated the prediction error in the test dataset using the Bray-Curtis dissimilarity. We emphasize that cNODE uses less information that Maynard's method, in the sense that the total biomass of each sample is unknown in this case.

The results of the comparison of Maynard's method and cNODE are shown in Fig. S5. We find that, for the above conditions used to construct the datasets, cNODE outperforms Maynard's method in both the training and test datasets. Crucially, note that cNODE was trained using only relative abundance measurements. We do not claim this results holds for all datasets, as there might be cases where the assumptions required by Maynard's method are exactly satisfied but none of the assumptions of cNODE are satisfied.

References

[1] Mike Innes. "Flux: Elegant machine learning with julia". In: *Journal of Open Source Software* 3.25 (2018), p. 602.

[2] Tian Qi Chen, Yulia Rubanova, Jesse Bettencourt, and David K Duvenaud. "Neural ordinary differential equations". In: *Advances in neural information processing systems*. Ed. by Samy Bengio, Hanna Wallach, Hugo Larochelle, Kristen Grauman, Nicolò CesaBianchi, and Roman Garnett. Montreal,Canada: Curran Associates Inc., 2018, pp. 6571–6583.

[3] Xavier Glorot and Yoshua Bengio. "Understanding the difficulty of training deep feedforward neural networks". In: *Proceedings of the thirteenth international conference on artificial intelligence and statistics*. Ed. by Yee Whye Teh and Mike Titterington. Sardinia,Italy: PMLR, 2010, pp. 249–256.

[4] Christopher Rackauckas and Qing Nie. "Differentialequations.jl – a performant and feature-rich ecosystem for solving differential equations in julia". In: *Journal of Open Research Software* 5.1 (2017).

[5] Diederik P Kingma and Jimmy Ba. "Adam: A method for stochastic optimization". In: *arXiv preprint arXiv:1412.6980* (2014).

[6] Sebastian Ruder. "An overview of gradient descent optimization algorithms". In: *arXiv preprint arXiv:1609.04747* (2016).

[7] Alex Nichol, Joshua Achiam, and John Schulman. "On first-order meta-learning algorithms". In: *arXiv preprint arXiv:1803.02999* (2018).

[8] FluxML. *MetaLearning.jl*. 2019. URL: https://github.com/FluxML/model-zoo/blob/master/contrib/meta-learning/MetaLearning.jl.

[9] John C Moore, Peter C de Ruiter, H William Hunt, David C Coleman, and Diana W Freckman. "Microcosms and soil ecology: critical linkages between fields studies and modelling food webs". In: *Ecology* 77.3 (1996), pp. 694–705.

[10] Jérôme Mounier, Christophe Monnet, Tatiana Vallaeys, Roger Arditi, Anne-Sophie Sarthou, Arnaud Hélias, and Françoise Irlinger. "Microbial interactions within a cheese microbial community". In: *Applied and Environmental Microbiology* 74.1 (2008), pp. 172–181.

[11] Richard R Stein, Vanni Bucci, Nora C Toussaint, Charlie G Buffie, Gunnar Rätsch, Eric G Pamer, Chris Sander, and Joao B Xavier. "Ecological modeling from time-series inference: insight into dynamics and stability of intestinal microbiota". In: *PLoS Computational Biology* 9.12 (2013).

[12] Charlie G Buffie, Vanni Bucci, Richard R Stein, Peter T McKenney, Lilan Ling, Asia Gobourne, Daniel No, Hui Liu, Melissa Kinnebrew, Agnes Viale, et al. "Precision microbiome reconstitution restores bile acid mediated resistance to Clostridium difficile". In: *Nature* 517.7533 (2015), pp. 205–208.

[13] Alison L Gould, Vivian Zhang, Lisa Lamberti, Eric W Jones, Benjamin Obadia, Nikolaos Korasidis, Alex Gavryushkin, Jean M Carlson, Niko Beerenwinkel, and William B Ludington. "Microbiome interactions shape host fitness". In: *Proceedings of the National Academy of Sciences* 115.51 (2018), E11951–E11960.

[14] Jonathan Friedman, Logan M Higgins, and Jeff Gore. "Community structure follows simple assembly rules in microbial microcosms". In: *Nature Ecology & Evolution* 1.5 (2017), p. 0109.

[15] Kelly S Ramirez, Jonathan W Leff, Albert Barberán, Scott Thomas Bates, Jason Betley, Thomas W Crowther, Eugene F Kelly, Emily E Oldfield, E Ashley Shaw, Christopher Steenbock, et al. "Biogeographic patterns in below-ground diversity in New York City's Central Park are similar to those observed globally". In: *Proceedings of the royal society B: biological Sciences* 281.1795 (2014), p. 20141988.

[16] Amir Bashan, Travis E Gibson, Jonathan Friedman, Vincent J Carey, Scott T Weiss, Elizabeth L Hohmann, and Yang-Yu Liu. "Universality of human microbial dynamics". In: *Nature* 534.7606 (2016), p. 259.

[17] Ophelia S Venturelli, Alex V Carr, Garth Fisher, Ryan H Hsu, Rebecca Lau, Benjamin P Bowen, Susan Hromada, Trent Northen, and Adam P Arkin. "Deciphering microbial interactions in synthetic human gut microbiome communities". In: *Molecular Systems Biology* 14.6 (2018), e8157.

[18] Christian Jost and Stephen P Ellner. "Testing for predator dependence in predator-prey dynamics: a non-parametric approach". In: *Proceedings of the Royal Society of London. Series B: Biological Sciences* 267.1453 (2000), pp. 1611–1620.

[19] Peter E Larsen, Dawn Field, and Jack A Gilbert. "Predicting bacterial community assemblages using an artificial neural network approach". In: *Nature Methods* 9.6 (2012), p. 621.

[20] Guangyu Zhou, Jyun-Yu Jiang, Chelsea J-T Ju, and Wei Wang. "Prediction of microbial communities for urban metagenomics using neural network approach". In: *Human Genomics* 13.1 (2019), p. 47.

[21] Daniel S Maynard, Zachary R Miller, and Stefano Allesina. "Predicting coexistence in experimental ecological communities". In: *Nature ecology & evolution* 4.1 (2020), pp. 91–100.

[22] Yandong Xiao, Marco Tulio Angulo, Jonathan Friedman, Matthew K Waldor, Scott T Weiss, and Yang-Yu Liu. "Mapping the ecological networks of microbial communities". In: *Nature Communications* 8.1 (2017), p. 2042.

[23] Jenny Tung, Luis B Barreiro, Michael B Burns, Jean-Christophe Grenier, Josh Lynch, Laura E Grieneisen, Jeanne Altmann, Susan C Alberts, Ran Blekhman, and Elizabeth A Archie. "Social networks predict gut microbiome composition in wild baboons". In: *eLife* 4 (2015), e05224.

[24] Jack C Yue and Murray K Clayton. "A similarity measure based on species proportions". In: *Communications in Statistics-theory and Methods* 34.11 (2005), pp. 2123–2131.

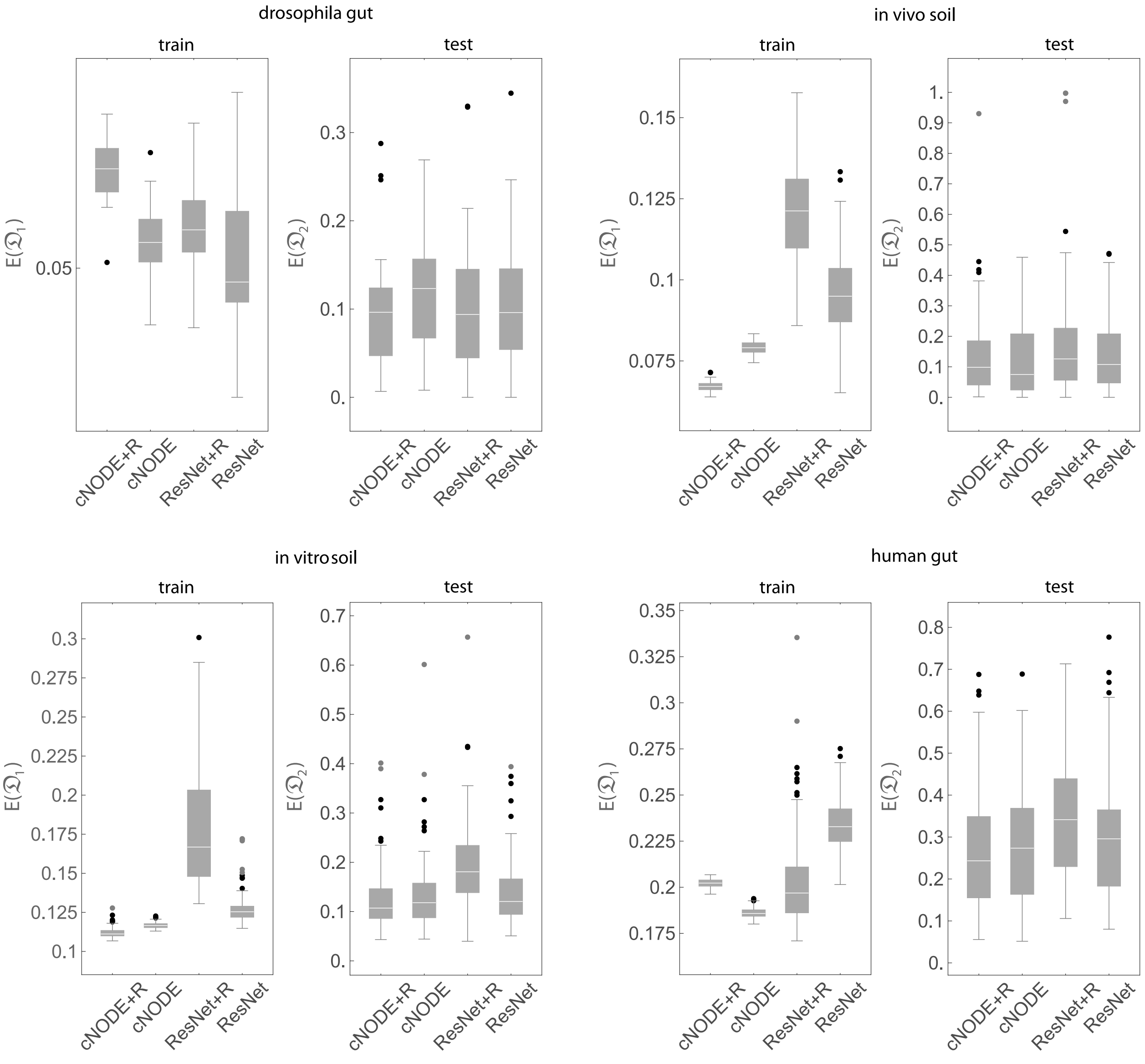

Supplementary Figure S1: **Performance of the ResNet and cNODE architectures for predicting compositions in experimental microbiomes.** Vertical axis denotes prediction error.

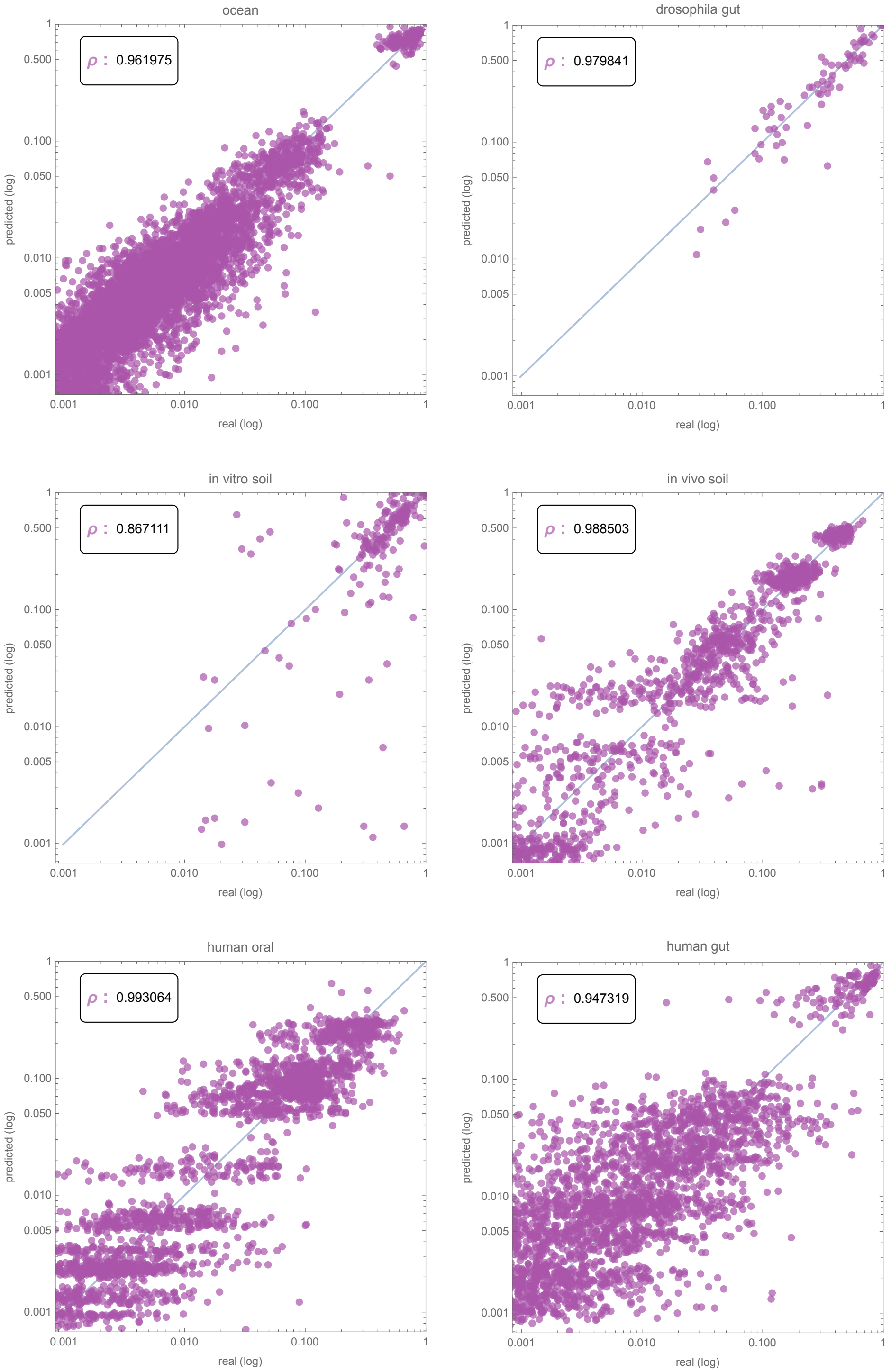

Supplementary Figure S2: **Correlation between true and predicted samples from experimental microbiomes.** Dots represent species abundances in log scale. Legends show the Spearman's rank correlation coefficient ρ.

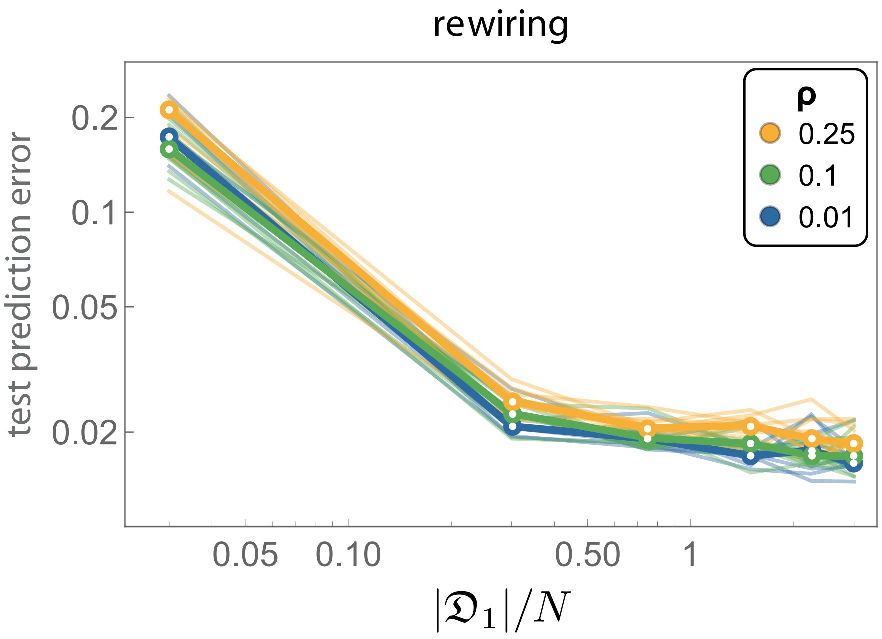

Supplementary Figure S3: ***In silico* validation of cNODE.** Results are for pools of $N = 100$ species with Generalized Lotka-Volterra (GLV) population dynamics. Lines show performance of cNODE for in-silico datasets with non-universal dynamics, produced by changing the species interactions in a sample-specific manner. The percentage of different interactions every sample is quantified by the value of ρ.

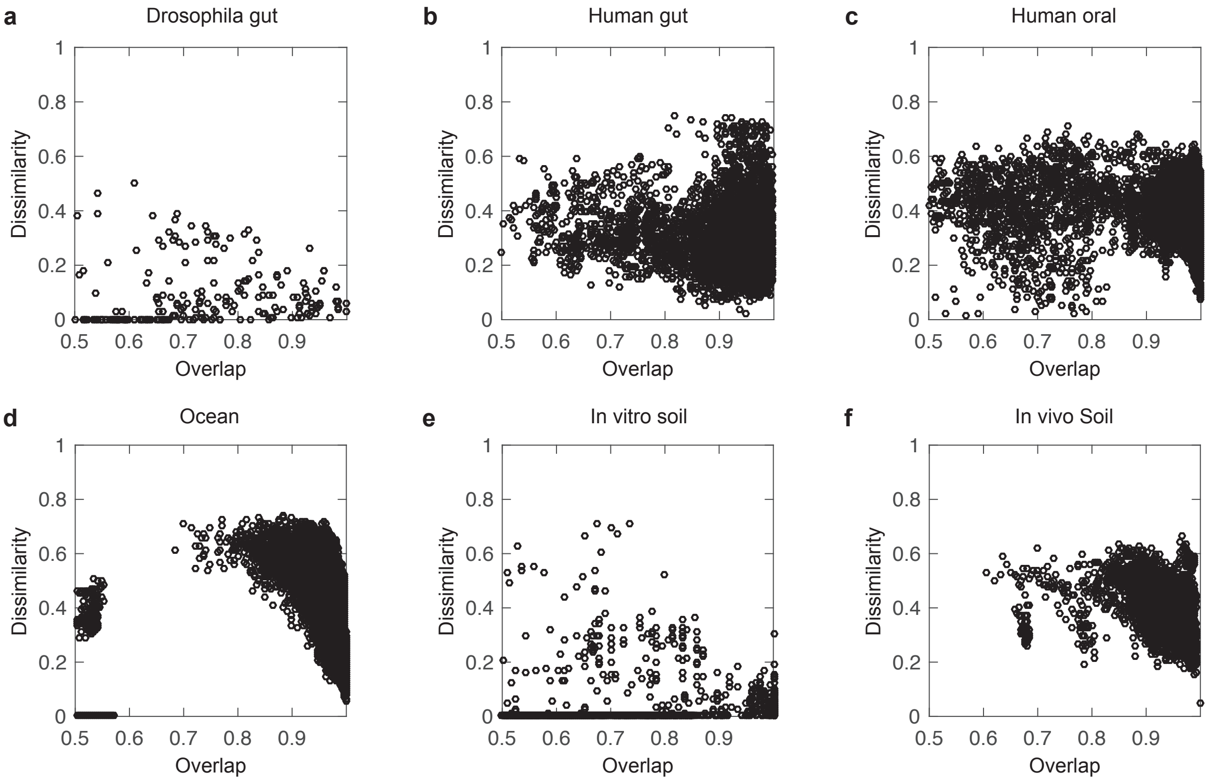

Supplementary Figure S4: **Dissimilarity-Overlap analysis of real microbiome datasets.** For the microbiome data of human gut, human oral, ocean, and in-vivo soil, the Dissimilarity-Overlap analysis was done at the genus level. For data from *Drosophila* gut and in-vitro soil, the Dissimilarity-Overlap analysis was done at the species level. Note that for those two datasets, due to the very low species richness ($N = 5$ and 8, respectively), there are many sample pairs sharing only one common species. These sample pairs have zero dissimilarity between the renormalized abundance profiles of the common species. This explains those data points with non-zero overlap but zero dissimilarity.

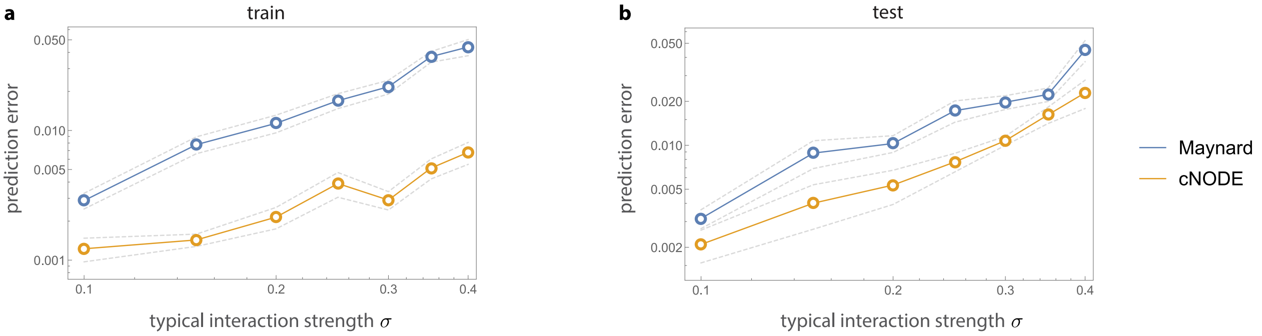

Supplementary Figure S5: **Prediction errors of Maynard et al.'s method[21] and cNODE.** For an *in silico* dataset of $N = 5$ species with universal dynamics and different typical interaction strength. Circles denote mean error for 10 repetitions, and gray shadows indicate standard deviation of the mean. **a.** Train dataset. **b.** Test dataset.

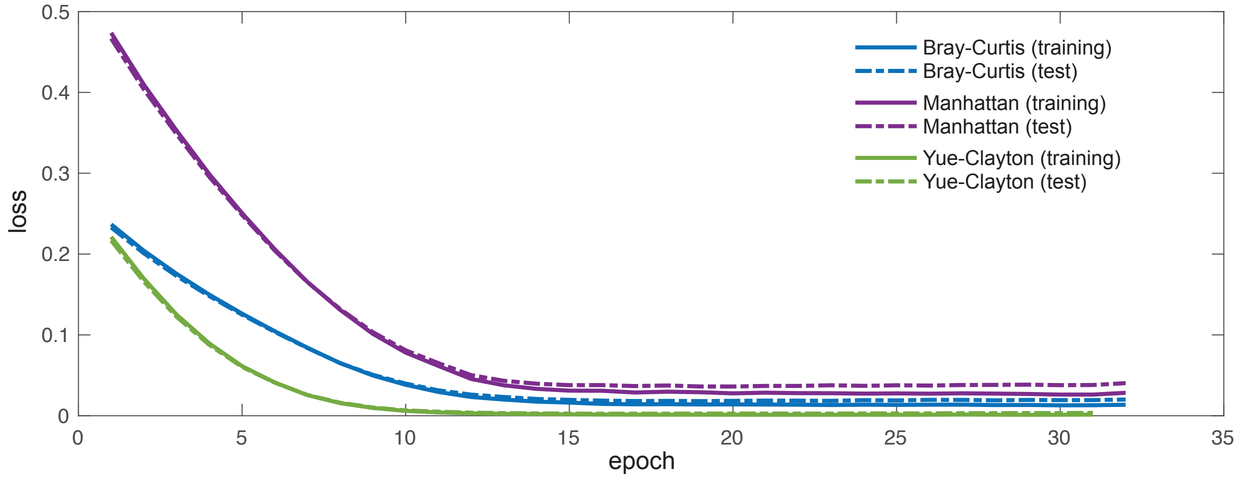

Supplementary Figure S6: **Performance of cNODE is quite robust to the change of dissimilarity or distance measure.** Results are for synthetic communities of $N = 100$ species generated by the Generalized Lotka-Volterra model with $\sigma = 0.1$ and $C = 0.5$. Prediction error was measured by the Bray-Curtis dissimilarity, Manhattan distance or Yue-Clayton dissimilarity[24] for training (solid) and test (dash) samples. Sample size for training, validation and test are 500, 100 and 400 respectively.
